# Cyclical depressurization degranulates platelets in an agonist-free mechanism of platelet activation

**DOI:** 10.1371/journal.pone.0274178

**Published:** 2022-09-15

**Authors:** Aaron J. Velasquez-Mao, Mark Velasquez, Moriel H. Vandsburger

**Affiliations:** 1 UC Berkeley–UCSF Graduate Program in Bioengineering, Berkeley, CA, United States of America; 2 Department of Bioengineering, UC Berkeley, Berkeley, CA, United States of America; Khalifa University of Science and Technology, UNITED ARAB EMIRATES

## Abstract

Activation of circulating platelets by receptor binding and subsequent coagulation events are defined by a well characterized physiological response. However, the growing prevalence of chronic kidney disease (CKD) and implication of platelet-released factors in worsening cardiovascular outcomes with hemodialysis warrant further investigation into the mechanobiology of platelet degranulation. The significant drops in pressure caused by high friction across the hemodialysis flow circuit present an overlooked platelet stimulant not involving immobilization as a driver for cytoskeletal rearrangement. In this study, platelets from healthy and dialysis (pre- and post-treatment) donors were cyclically depressurized in static suspension to measure changes in physiology by integrin α_IIb_β_3_ activation and surface P-selectin expression. The progressive increase in CD62P with no changes in PAC1 over pressure-cycling duration regardless of uremia signifies that hydrostatic depressurization involves a novel agonist-free mechanism leading to platelet degranulation as a unique case in which CD62P and PAC1 do not interchangeably indicate platelet activation. Subsequent stimulation using ADP further suggests that sustained depressurization regimens desensitize integrin α_IIb_β_3_ activation. Variability in platelet response caused by uremia and CKD are observed by elevated baseline PAC1 in pre-dialysis samples, PAC1 retention after ADP exposure, and maximum CD62P with ADP independent of pressure. Theory for hydrostatic pressure-induced degranulation circumventing integrin-initiated signal transduction is here presented based on the Starling Equation.

## Introduction

Platelets play a broad role beyond hemostasis as circulating sources of tissue regulatory factors [1]. The contribution to platelet dysfunction by blood filtration therapies like hemodialysis is widely attributed to filter material reactivity and high shear beyond uremia [2, 3]. However, these mechanisms activate platelets by receptor adhesion, whether by defective GPIb and GPIIb/IIIa [4], hydrophobic capture [2], or rolling immobilization to RGDS sites on unfurled vWF [5]. Recent hemodialysis findings link chronically pro-fibrotic circulations to increasingly differential platelet granule secretory kinetics [6], amplifying the need to interrogate the drivers and mechanisms of platelet degranulation.

Examining dialysis, the use of hollow microtubes and ultrafiltration produces a flow circuit characterized by rapid and repetitive pressure drops. While receptor-driven signal transduction is well mapped, the effects of non-native pressure cycling present a potential internally-driven alternative that enhances pathological degranulation. Ronco et al measured frictional pressure drops across 200μm diameter hollow fiber dialyzers of up to 200mmHg at a 500mL/min blood flow rate [7]. Interestingly, existing studies on platelets subjected to pressure-based stimulation in isolation are limited. In decompression sickness platelets have heightened reactivity linked to sudden circulatory decompression [8], while the role of platelets in high altitude thrombus formation has long been a topic of debate [9, 10]. Hydrostatic decompression induces platelet aggregation during reductions from both atmospheric pressure and high pressurization irrespective of oxygen partial pressure [11], while positive pressure inhibits aggregation [12]. Elsewhere, hydrostatic pressure directly regulates eukaryotic cell volume, cortical tension, and intracellular ion concentration [13]. The application of positive pressure inhibits exocytosis in patch-clamped nerve cells [14], induces volumetric contraction by cytoskeletal rearrangements [15], and causes ion transport [16] including by effects on volume-regulated channels [13]. For platelets, whose behavior is intricately linked to geometry, sphericalization and cytoskeletal expansion characterize the first steps of activation preceding coagulation [17]. Thus, expansive depressurization, particularly of a cyclical nature, may drive platelet activation by influencing the cytoskeleton, ion transport, and exocytotic surface expansion.

In these experiments, platelets are subjected to a more intense depressurization of 460mmHg to establish an upper bound of effects from this isolated stimulus. To exaggerate the repeated acclimation and stimulation characteristic of dialytic recirculation, chamber depressurization occurred periodically over 0.7s at maximum pump evacuation followed by instantaneous re-pressurization to atmospheric levels and 0.7s of rest. Platelet activation was measured by detection of activated GPIIb/IIIa (PAC1) and P-Selectin (CD62P), which is localized to internal granules and externally presented upon degranulation. Because of differences measured in these historically interchangeable biomarkers of platelet activation [18], expression was also measured as a function of ADP exposure for pressure-stimulated and unstimulated (0 minute) samples. Experiments were repeated on platelets from dialysis patient donors before and after hemodialysis to measure differences in clinical settings. Common expression trends reveal a novel and universal basis for pressure-driven degranulation distinct from shear-based activation, while contrasting population baselines substantiate response variability owing to uremic dysfunction. Depending on the proteomic and transcriptomic contents of circulating platelets, this could exacerbate platelet contributions to fibrotic pathologies and cardiovascular dysregulation in vulnerable patient demographics [6].

## Methods

### Blood collection & platelet isolation

Written informed consent was obtained from all participants under protocol number 2017-04-9810 approved by the IRB at University of California, Berkeley. Hemodialysis blood samples were collected immediately before vascular access and after tubing removal from four patients at DaVita Oakland. All patients were dialyzed using NIPRO single-use, hollow-fiber ELISIO filters. Four control blood samples with no history of smoking, drug abuse, current medications, or comorbidities were collected at the University of California, Berkeley Tang Center. Table 1 summarizes subject demographics. All samples were collected in ACD-A Vacutainers and processed immediately by centrifugation at 200g for 10min to collect the top 90% of platelet-rich plasma (PRP). Collected PRP was centrifuged again for 5min at 200g to remove remaining erythrocyte and leukocyte contamination and gently pipetted into a 60mL plastic syringe in a BSL2 biosafety cabinet for immediate use. Participants provided informed consent under protocols approved by the U.C. Berkeley IRB and in compliance with the Declaration of Helsinki.

**Table 1:** Subject demographics.

Control demographics (n = 4)	
Age, years—median (range)	25 (25–26)
Gender ratio—[male/female]	4/0
Patient demographics (n = 4)	
Age, years—median (range)	69 (46–79)
Gender ratio—[male/female]	3/1
Ethnicity	
African American	2
Asian American	1
Caucasian	1
Prior time on dialysis, months—median (range)	24 (3–96)
Cause of renal failure	
Hypertension	3
Diabetes mellitus	1
Comorbidities	
Anemia	2
Hyperlipidemia	1
CHF	2
Hypothyroidism	1
Medication	
Lipid-lowering drugs	2
Platelet inhibitor (non-P2Y12)	2
RAS Inhibitors	1
None	0

### Cyclical depressurization & sample collection

**Fig 1:**
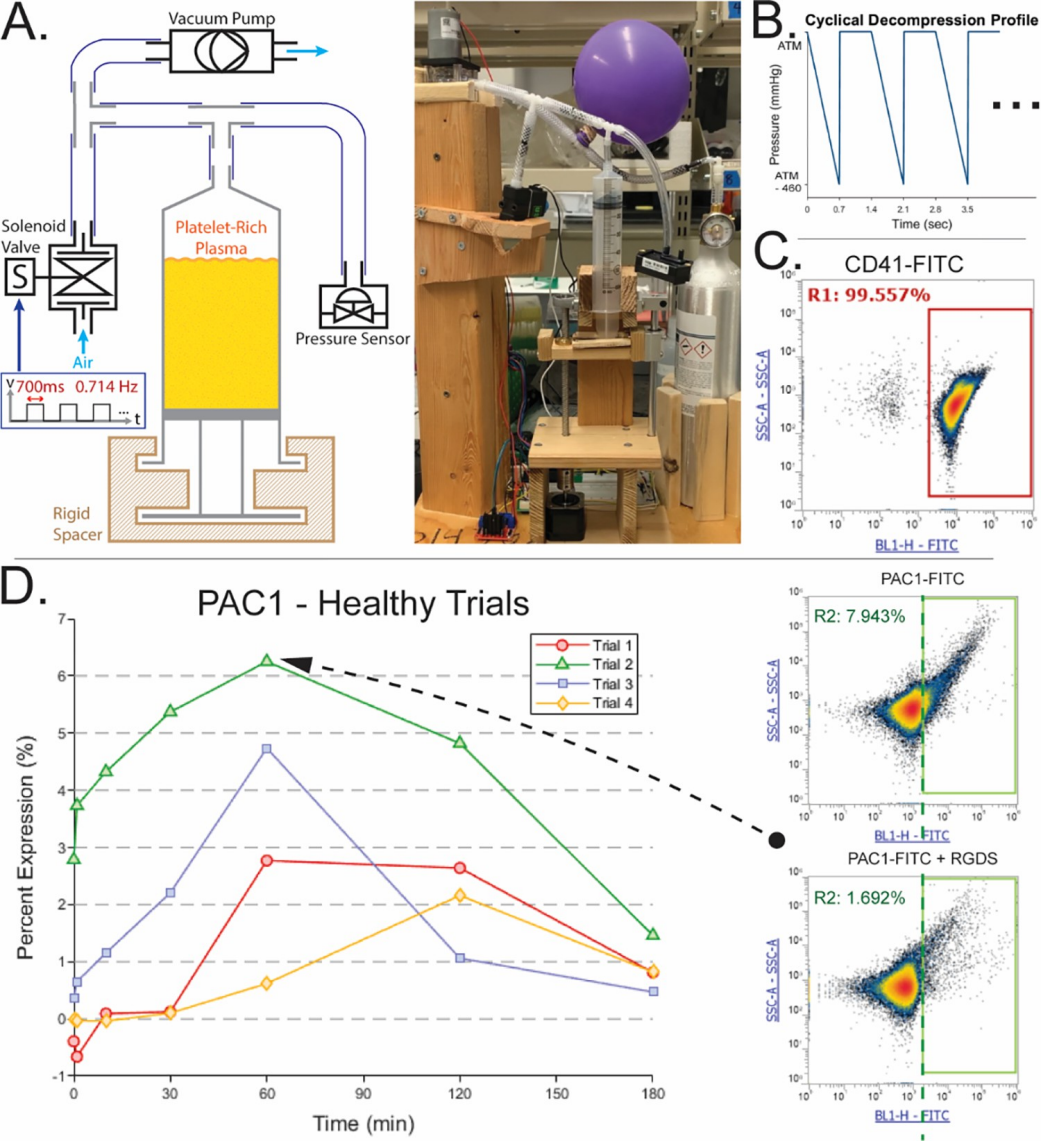
Experimental setup. (**A**) Machine for static depressurization of platelets suspended in plasma within a syringe. (**B**) Hydrostatic pressure profile experienced by platelets involving linear evacuation of 460mmHg from atmospheric pressure over 700ms followed by instantaneous release at 50% duty cycle. (**C**) CD41 positive gating using FITC isotype control indicates >99% pure platelet populations across samples. (**D**) Flow cytometry measurement example of PAC1 by subtraction of RGDS control.

The PRP-loaded syringe was evacuated isochorically by 460mmHg using a rigid spacer and 12V/12W diaphragm vacuum pump under constant maximum operation (Fig 1A). The chamber was re-pressurized to atmospheric using a 12V solenoid valve given an electrical cycle of 700ms impulse followed by 700ms release (Fig 1B). Chamber pressure was measured by a vacuum transducer (Omega, PX141) elevated to water-level. Valve, pump, sensor, and stepper motor-operated spacer were controlled by NI myDAQ using LabVIEW. PRP was collected by gentle extrusion from the syringe at 0min (no stimulation) through 180min of cyclical depressurization into sterile polypropylene and left at dark room-temperature (RT) until final time-point collection.

### Flow cytometry & controls

Upon final collection, sub-samples were siphoned and exposed to ADP at 2.5 x 10^-4^M for 5min at RT. Then, antibodies for CD41 (Thermo, MA180666), PAC1 (Thermo, BDB340507), and CD62P (Thermo, 12-0626-82) were added 1:2 to 5μL PRP at RT for 15min in a 96U plate, followed by addition of paraformaldehyde in calcium-free saline to 1%. Arg-Gly-Asp-Ser (RGDS) (Sigma, A9041) was added to a separate PAC1 stain mixture to 2 x 10^-2^M prior to platelet labeling as a competitive inhibitor to subtract nonspecific binding (Fig 1D). Isotype controls for FITC (Thermo, 11-4714-81) and PE (Thermo, 12-4714-82) were used for each sample to subtract nonspecific binding. Single-plex fluorescence was measured on an Attune NxT Flow Cytometer immediately after staining. The same fluorescence gates were used across all trials.

### Statistics

Significantly different means were identified amongst test groups by Friedman’s ANOVA followed by Dunn & Sidák’s multiple comparisons test. Measurement distributions on figures were compared by two-sample t test, assuming unequal variance and two tails. Linear dependencies were assessed by Pearson correlation. Significance in all cases was defined by p ≤ 0.05. Figure error bars represent 95% confidence intervals.

## Results

**Fig 2:**
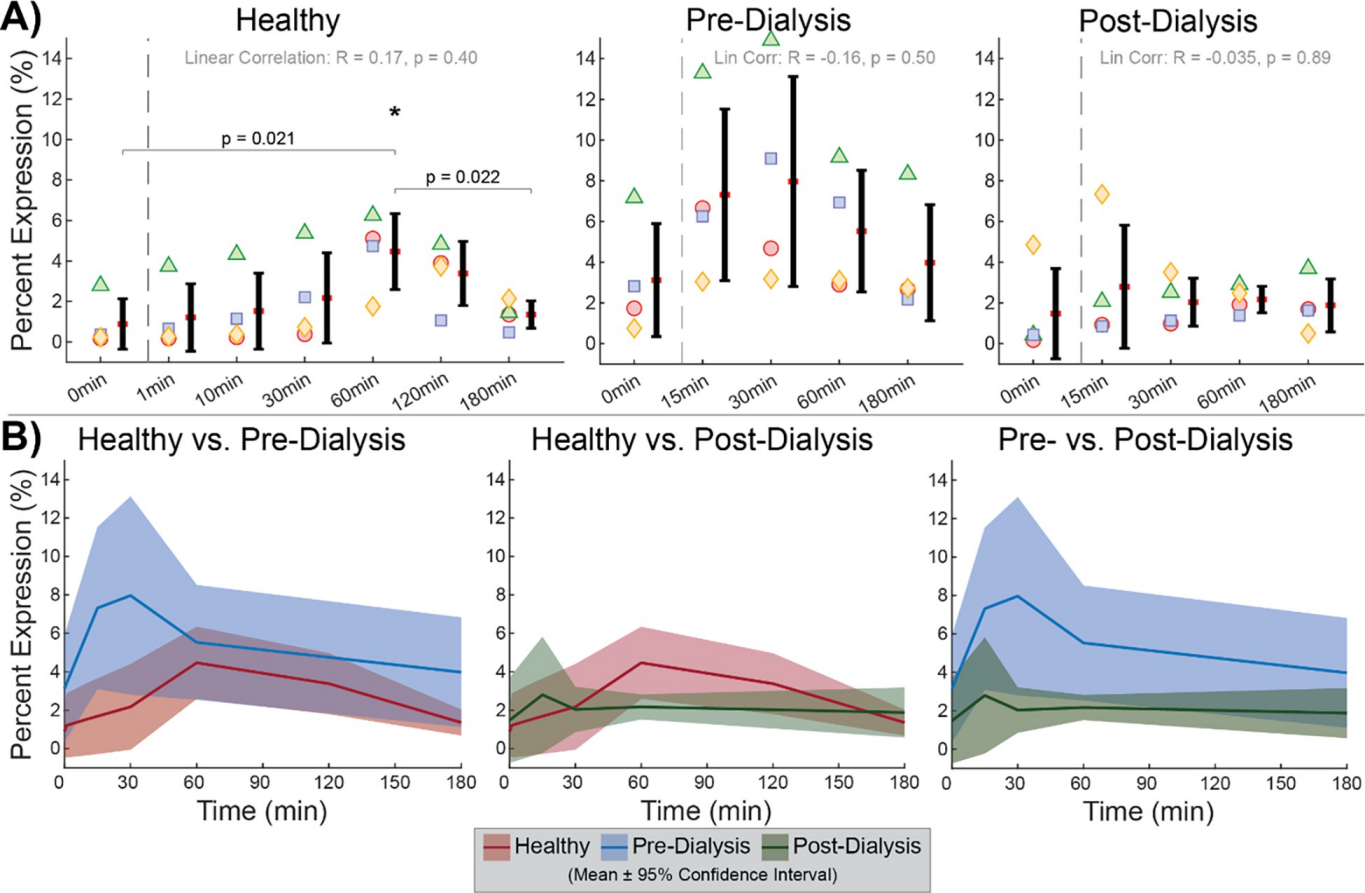
Platelet PAC1 expression. (**A**) No linear correlations observed in PAC1 as a function of pressure-cycling duration within healthy, pre-dialysis, and post-dialysis test groups. (**B**) No significant differences observed in percent expression between test groups at any time point.

**Fig 3:**
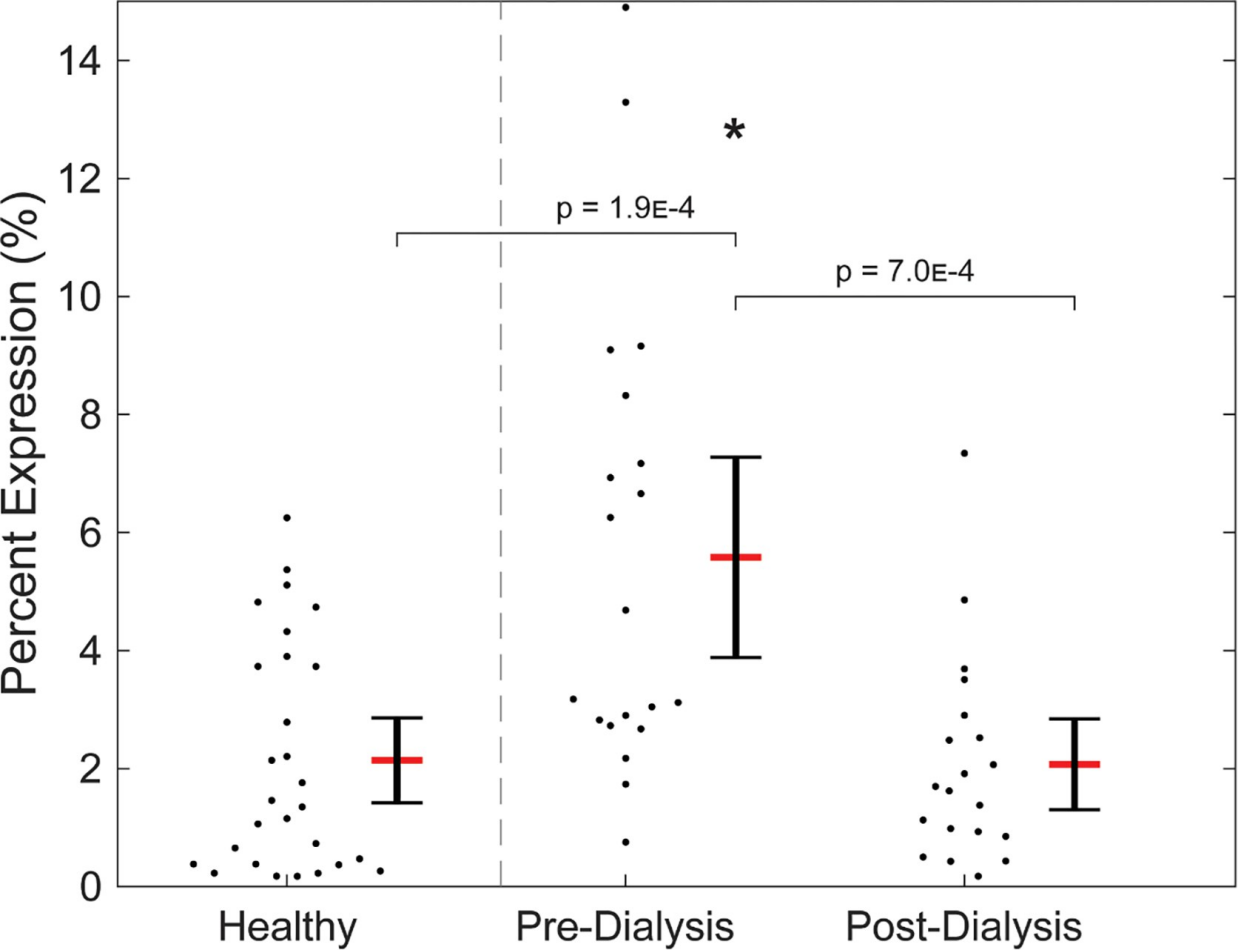
Test group-aggregated PAC1 expression. Pre-dialysis platelets had elevated expression compared to healthy and post-dialysis platelets, which had similar ranges.

Pure platelet populations across trials were confirmed by unimodal forward-scatter (FSC) against side-scatter (SSC) density and > 99% CD41 expression (Fig 1C). Cyclical depressurization alone yielded no response in platelet PAC1 expression (Fig 2). No linear trends were observed within healthy, pre-dialysis, and post-dialysis test groups (Fig 2A). No differences were found between any two test groups at any time point (Fig 2B). A possible transient difference in receptor rate of change is observed by significantly elevated PAC1 expression at 60min compared to 0min and 180min in healthy trials (Fig 2A). However, no time point had a mean significantly different from any other time point for healthy (p = 0.062), pre-dialysis (p = 0.35), or post-dialysis (p = 0.90) trials by Friedman’s ANOVA. In the absence of a universal PAC1 trend observed as a function of pressure-cycling duration, expression data was aggregated by test group across all time points to assess baseline differences attributable to population (Fig 3). Healthy and post-dialysis samples had nearly identical ranges (2.1±1.9 vs 2.1±1.8, mean±SD), while pre-dialysis measurements were significantly elevated and significantly more varied (SD = 3.9). Friedman’s ANOVA identified a significantly different group mean (p = 2.7E-5), while Dunn & Sidák’s multiple comparisons test pinpointed pre-dialysis measurements as significantly different from healthy (p = 9.0E-5) and post-dialysis measurements (p = 2.2E-4). Thus, overall PAC1 elevation in pre-dialysis samples may be attributed to uremia, while baseline expression levels in the same dialysis patients are comparable to a healthy range after uremic clearance.

**Fig 4:**
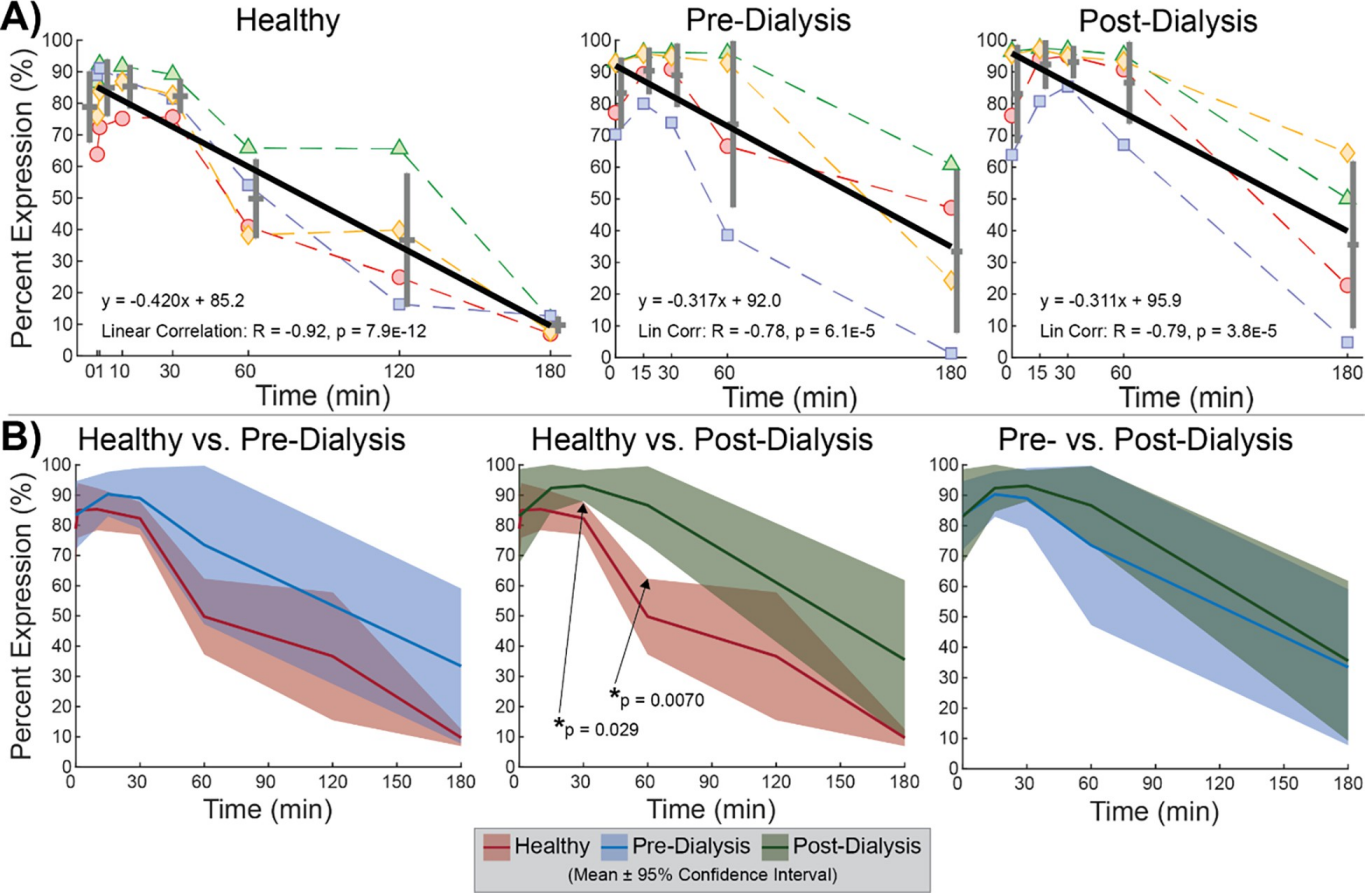
PAC1 expression for samples subsequently stimulated with ADP. (**A**) Expression correlated negatively to pressure-cycling duration in all test groups, with healthy platelets diminishing fastest. (**B**) Faster reduction in healthy platelet PAC1 expression observed by significant differences in percent expression at middle time points.

When subsequently stimulated with ADP, platelets reliably decreased in PAC1 expression with increasing duration of pressure-stimulation. Negative linear correlations were detected in all test groups (Fig 4A). However, healthy platelets demonstrated a greater reduction in expression overall (R = -0.92) compared to pre-dialysis (R = -0.78) and post-dialysis (R = -0.79) platelets. In the healthy test group, platelets at 60min (p = 0.015), 120min (p = 0.014), and 180min (p = 2.4E-5) expressed PAC1 at significantly lower levels compared to pressure-unstimulated platelets at 0min. In comparison, pre-dialysis (p = 0.013) and post-dialysis (p = 0.022) platelets expressed significantly lower levels of PAC1 only after 180min of pressure cycling. The more rapid reduction in PAC1 expression in healthy platelets with longer pressure-cycling duration preceding identical exposure to ADP compared to dialysis platelets is further reflected in significantly lower expression at 30min and 60min compared to the post-dialysis population (Fig 4B).

**Fig 5:**
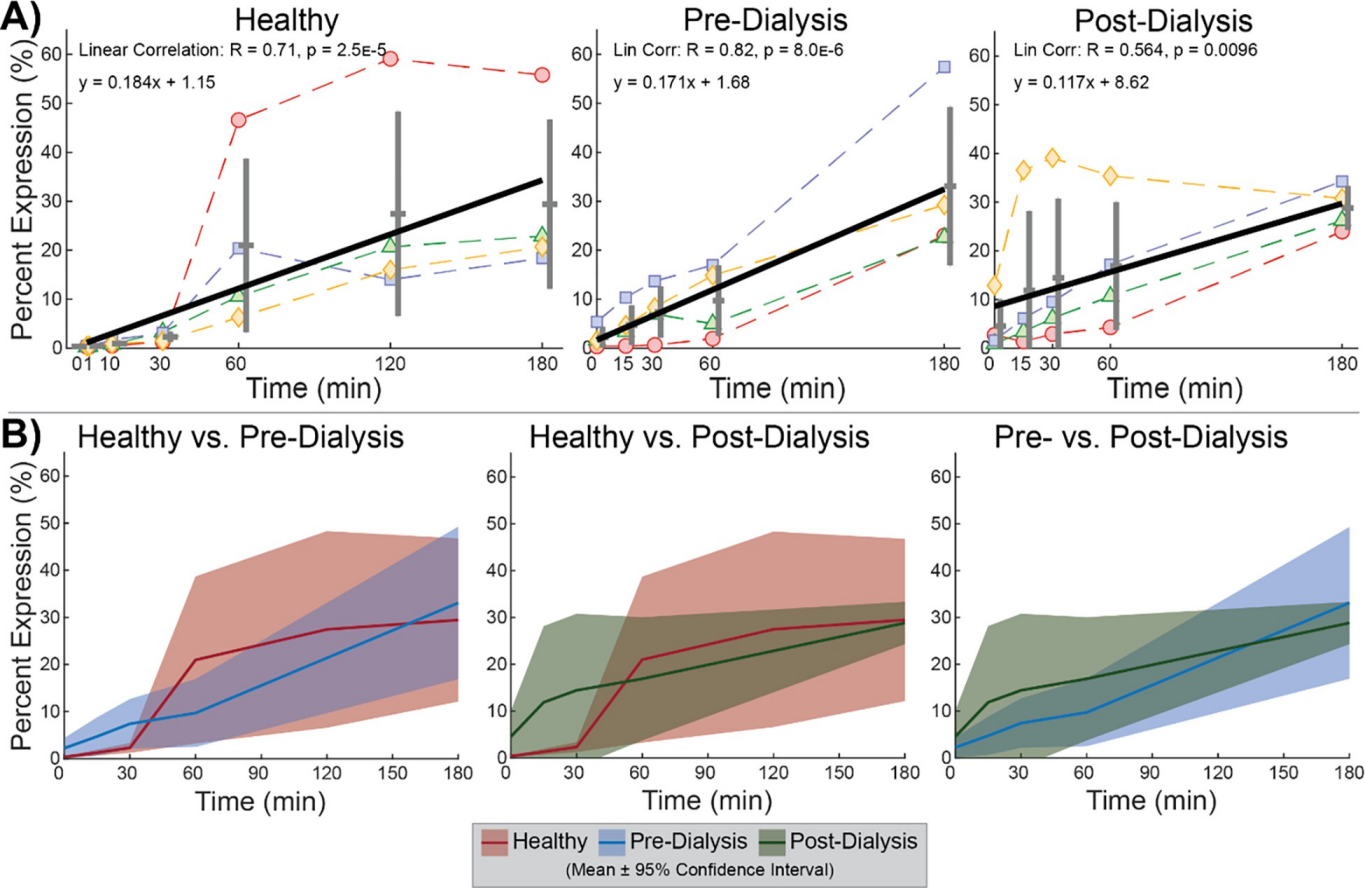
CD62P expression. (**A**) Expression correlated positively to pressure-cycling duration in all test groups. (**B**) No significant differences observed in percent expression between test groups at any time point.

**Fig 6:**
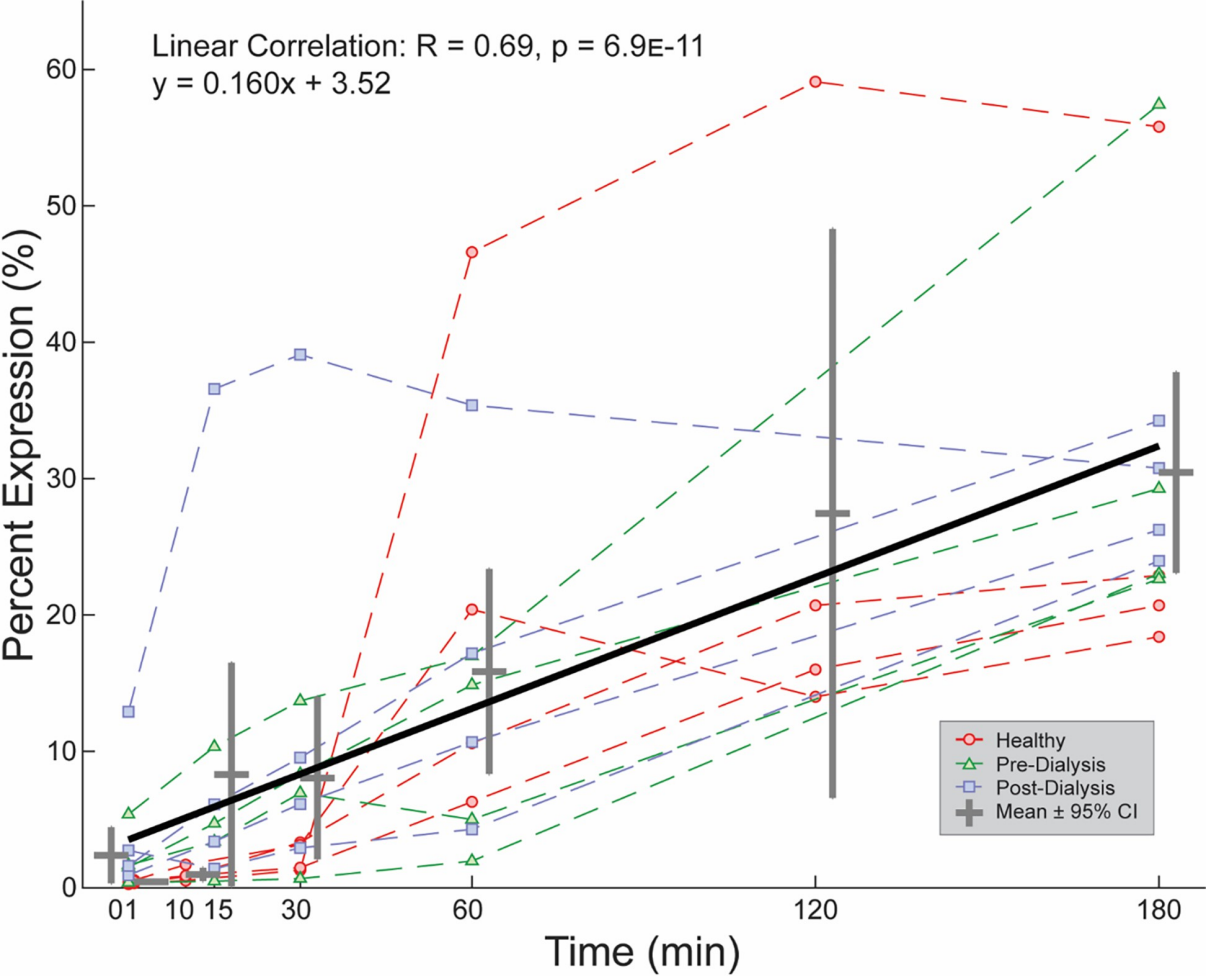
**Total aggregated CD62P expression.** Expression universally increases with pressure-cycling duration, regardless of uremic state.

Unlike PAC1, CD62P unilaterally increased in expression with cyclical depressurization. Positive linear trends were detected in all test groups (Fig 5A), with no differences measured between test groups at any time point (Fig 5B). These trend consistencies led to the aggregation of expression data across all test groups to measure overall CD62P behavior as a function of pressure-cycling duration (Fig 6). Increased statistical strength (p = 6.9E-11) signifies that CD62P increases in expression with pressure stimulation as a universal platelet response that is agnostic to uremia. Because CD62P is originally stored in differentiated granules within platelets, these data support that repetitive depressurization induces degranulation.

**Fig 7:**
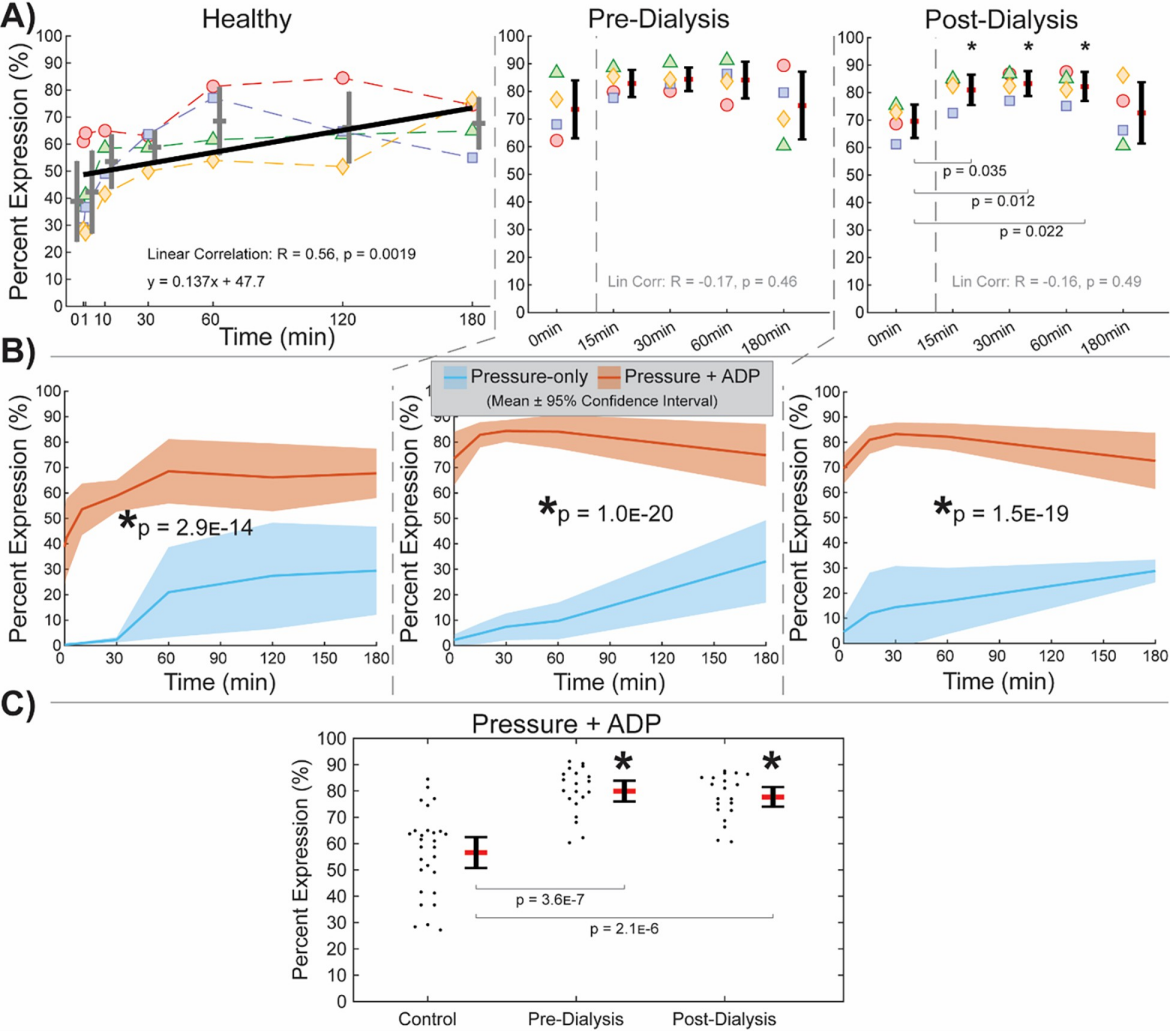
CD62P expression for samples subsequently stimulated with ADP. (**A**) A positive trend in healthy platelets indicates additive effects, while no trend in pre- or post-dialysis platelets is likely from heightened sensitivity to stimulation. (**B**) ADP increases expression for all test groups at all time points. (**C**) Healthy platelets have lower baseline expression levels than both pre- and post-dialysis platelets.

When subsequently stimulated with ADP, platelets expressed CD62P in greater proportion, with behavioral discrepancies observed between healthy and dialysis samples. A positive correlation was observed in the healthy test group, while no significant trend could be fitted to pre-dialysis or post-dialysis measurements (Fig 7A). However, pressure-and-ADP-stimulated platelets expressed greater CD62P at all durations of pressure stimulation compared to pressure-only stimulated platelets from the same test group (Fig 7B). Thus, while for healthy platelets ADP and depressurization may have additive effects on degranulation, dialysis patient platelets may have heightened sensitivity to both pressure and ADP. The latter is supported by significantly higher expression levels in ADP-only (0 minute) stimulated platelets for both pre-dialysis (p = 0.0098) and post-dialysis (p = 0.0097) samples. Furthermore, significant elevation in middle time points for post-dialysis samples may corroborate additive interactions between pressure and ADP on degranulation, while the absence of significant differences between any time points in pre-dialysis samples may indicate a dependency in sensitivity on uremic state. Comparing test group aggregates confirms elevated baseline expression levels in dialysis samples (Fig 7C). Friedman’s ANOVA identified a significantly different group mean (p = 2.1E-9), while Dunn & Sidák’s multiple comparisons test pinpointed healthy measurements as significantly different from pre-dialysis (p = 3.3E-8) and post-dialysis measurements (p = 4.0E-7).

## Discussion

Cyclical depressurization induced platelet degranulation without activating the GPIIb/IIIa integrin complex in a unique and unexplored mechanism of platelet stimulation. While CD62P increased linearly with duration of pressure cycling, PAC1 expression remained at base unstimulated rates. In comparison, when stimulating platelets by shear stress, both CD62P and PAC1 interchangeably predict platelet activation [18]. Interestingly, co-stimulation with ADP additively enhanced CD62P expression while decreasing PAC1 expression. When stimulated using ADP alone, platelets have been shown to retain PAC1 expression for up to 8hr [18]. Taken together, these data suggest that depressurization circumvents receptor signal transduction to induce exocytosis and further that this mechanism desensitizes platelets to chemical stimuli. In hemodialysis, this may contribute to bleeding disorders [2] while still exacerbating the circulation of platelet factors [6].

While CD62P profiles were universal, population-level differences observed between healthy, pre-dialysis, and post-dialysis PAC1 measurements indicate effects attributable to uremia and chronic kidney disease (CKD). Pre-dialysis platelets exhibited overall PAC1 elevation compared to healthy and post-dialysis platelets whose profiles were equivalent, indicating a baseline state of hyperactivity in dialysis patients that is corrected with uremic clearance and platelet renewal. Further, the significant early increase in paired pre-dialysis platelet PAC1 expression could signify a heightened sensitivity to pressure shock. However, the “acclimation” observed by a significant negative rate of change at 60min to average unstimulated levels may imply that perturbations on PAC1 caused by depressurization are not sustained, regardless of uremic state. Examining the combinatorial effects of ADP on top of pressure, both pre- and post-dialysis platelets showed a slower decline in PAC1 expression over time compared to healthy platelets considering comparable ranges at 0 minutes of pressure stimulation between all groups. As pressure-inhibition of GPIIb/IIIa may be beneficial in preventing capillary bed plugging [19, 20], inclined retention of the activated integrin after agonist exposure demonstrates a troubling adaptive possibility in which CKD patient platelets remain preferentially adherent for longer.

Study limitations include low donor count, higher pressure drop used than experienced across hemodialysis, the use of a single pressure profile, number of platelet activation reporters, and additional agonists used. The experimental pressure drop of 480mmHg is over two-fold higher than that experienced by platelets during hemodialysis, though remains relevant as a benchmark for the progression of blood ultrafiltration technology [7]. Additionally, recruitment protocols at University Health Services and DaVita restricted accessible demographics, resulting in an age mismatch between healthy and dialysis donors. Future studies should probe the effects of varying pressure drop rate and frequency to establish bounds of safe usage. Further investigation of depressurization effects on phosphatidylserine and clotting factor binding would better characterize whether the same downstream procoagulant phenotype as from shear is produced [19, 21]. Similarly, sensitivity to other agonists including thrombin would round out the resulting coagulative profile [1, 17].

## Theory

The Starling Equation describes an outward volumetric flux, J, across a membrane driven by hydrostatic pressure P and osmotic pressure π.

$$J_{v}=L_{p}S(\left[P_{in}-P_{out}]-\sigma [\pi_{in}-\pi_{out}\right]),$$

where L_p_, S, and σ are constants.

A sudden reduction in P_out_ by external hydrostatic depressurization would generate a solvent convective force outwards unless met with a net ion influx, increasing π_in_, thereby expanding cortical volume. In these experiments, no population-level changes in platelet diameter were evident by forward scatter distributions, so any potential volumetric changes to pressure shock were not sustained after stimulation. Thus, either absorptive ion influx was followed by equivalent contracting ion outflux upon vacuum release, or filtrative solvent outflow caused localized forces at the platelet membrane followed by reabsorption. In both cases, the cytoskeleton is strained, and both geometric rearrangements are evident in the classic clotting response. In fact, in response to agonist-induced activation, platelets first become spherical by the influx of calcium and secondly hydraulically generate podia [17]. While neither platelet shape nor volume were actively tracked throughout the applied pressure cycle, the feasibility of inducing these same swelling and protruding forces without a surface receptor agonist may explain the here-mentioned observations of downstream degranulation by CD62P presentation without initiation by integrin signal transduction measured by PAC1.

The theorized cortical forces across platelet membranes may be symptomatic of other non-contact platelet stimuli beyond the repetitive frictional pressure shock characteristic of hemodialytic filtration. Taking the other driver of flux from the Starling Equation, osmotic shock likely also induces platelet degranulation by the same principles. Early studies reported platelet morphological expansion [22], aggregation [23], and free ion flux [24] in both hyper- and hypotonic solutions with implications on blood storage. Considering the drastic shifts in osmolarity experienced in the renal medulla and procoagulant platelet phenotypes observed in representative hyperosmotic solutions [21], agonist-free platelet degranulation may contribute to worsening CKD in dialysis patients exhibiting pro-fibrotic platelet disposition [6].

## Supporting information

S1 Graphical abstract (TIF)
