# Supplementary figures and images for: Cyclical depressurization degranulates platelets in an agonist-free mechanism of platelet activation
Source: PLoS One. 2022 Sep 15;17(9):e0274178. doi: 10.1371/journal.pone.0274178 (PMC9477271; doi:10.1371/journal.pone.0274178)

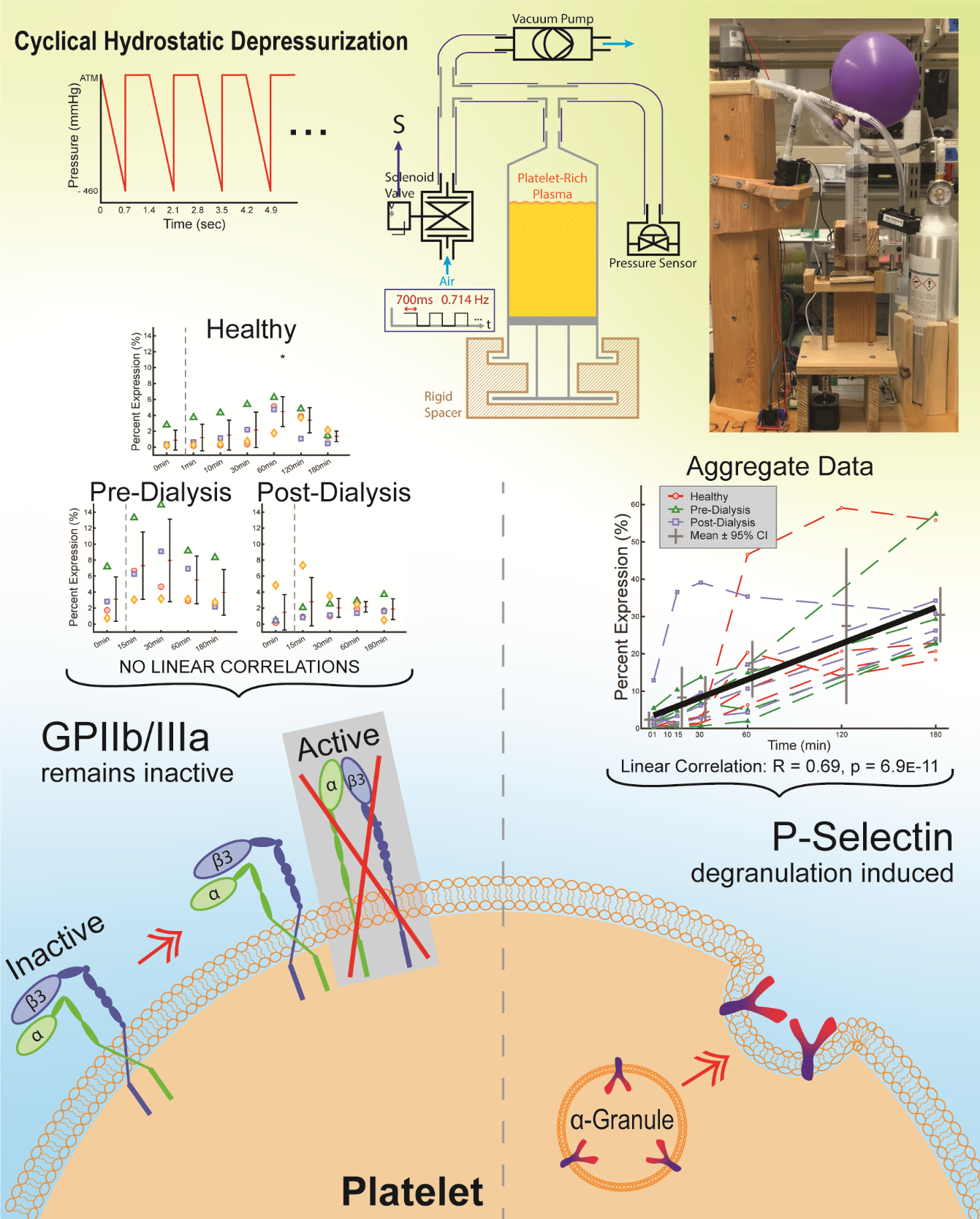

Supplement: S1 Graphical abstract — (TIF) [file pone.0274178.s001.tif]
